# Editorial Chit-Chat

**Published:** 1877-10

**Authors:** 


### Editorial Chit-Chat.

"SWEET FORGET ME NOT," is a very pretty little song, published by F. W. Helmick, of Cincinnati, O.

AN ARTIST.—Mr. Jas. B. Chandler, of Philadelphia, an artist of considerable distinction, has opened a studio in the Surgical Institute, and is making some fine pictures of dogs' heads.

B. BROWNE—He who has heretofore written so acceptably upon children and their ways—has an article in store on "ONLY A BOY," which came too late for this number. We are saving it for our next.

PLEASANTON'S HUMBUG. — We hear nothing more of the blue-glass cure. And has it subsided so soon? Would it not be interesting to know just how many fools purchased blue-glass, upon the recommendation of that inspired idiot?

OUR ADVERTISERS.—Read our advertisements. They are all of reliable houses, as will be seen. You may always rely upon the respectability of any house represented in our journal. None others are admitted upon *any* terms. In addressing advertisers, please mention THE BISTOURY.

GARDEN STATUES.—When you return from your Club—or Board of Education meeting—after midnight, and find your yard full of nodding, ghostly statues, be not afraid, closer inspection will reveal the flowers and shrubs, covered with papers and sheets to protect them from the frosty nights.

THE TELEPHONE.—This wonderful instrument has been sufficiently perfected to be in practical use. A Mr. Williams, of Boston, has a line connecting his place of business in that city with his residence at Somerville, three miles distant. Mr. Williams says that by his line, conversation can be carried on nearly as well as if those conversing were in the same room.

FLORISTS.—We have discovered that florists differ as widely over the treatment of plants as do doctors over their patients. We have found it a pretty good rule, so far, to do just the contrary to what florists tell you, in the management of plants. You will succeed much better. Do not take the same advice with regard Doctors, however,—that would be fatal.

PAY YOUR SUBSCRIPTION.—Many of our subscribers are in arrears with their subscriptions to THE BISTOURY. Better pay up, or you will find yourself without the journal. We print only our regular edition, supplying all new subscribers (as far as we can) by cutting off the dead-heads.

BRIGHAM YOUNG. — He died—suffered *rigor mortis*, on August 29th, from that homely disease,—cholera morbus. Now, had Brigham lived a reasonable while longer, it would have been quite within the possibilities, that he might have expired from *asphyxia*, or, at least, dislocation of his *cervical vertebræ.*

AUTUMN LEAVES.—Collect the beautifully hued autumn leaves as they drop upon the ground. Place them between the leaves of a large book, and under some heavy weight. Remove them once a week, and place your book in the sunlight, that all moisture may be removed. Leaves treated in this manner will retain their colors, will not curl, and make beautiful adornments for the parlor. The woodwork of an arch leading to a bay-window, when covered with leaves and ferns, is a beautiful object to look upon.

A GHOST.—It was in the morning early, just as the gray dawn began to reveal the trembling leaves upon the prairie queen that climbed the trellis near our bed-room window, when we were awakened from a deep sleep by a screech that set our cerebo-spinal axis of nerves in a quiver. We sat bolt upright in bed and listened,—listened and watched,—when suddenly a plaintive strain came stealing through the open door,—so full of love, anguish or terror, (bless us if we could determine which) that sent a thousand imaginings through our soul! Was the baby sick—could she be dying and her mother sleeping the while, unconscious of the peril of the babe? Terrible thought! Just as we were about to set our uncovered limbs in motion toward the nursery door, another wail greeted our ears, like the shriek of a dying maniac, then lulled into the gentle laughter of a sleeping babe, (as when angels are said to be whispering in its ears); then rose again to the giggle of a frolicsome school-girl, increasing in intensity and varying in tone until a very pandemonium of sounds greeted our astonished ears, bouncing us in terror from our bed to the chamber floor. Suddenly, the sounds lulled again into a soothing harmony, growing slower and fainter, until its peaceful note died in the dim distance of our parlor, whence we had traced it, to find our second son practising his fifth lesson upon the violin.

THE TURKS.—"They fought like Turks," has been an expression much in use, to indicate determination, pluck and bravery. All these qualities seem to be the attributes of the followers of the crescent, on the Danube. Who would have supposed those Turkish heathen would or could resist the sturdy Russians so long? Truly they *do* "fight like Turks," and the end is not yet.

---

JIM CROW.—Jim has developed a new accomplishment since our last issue. He is now devoting his spare moments to pugalistic encounters. We will wager a cookie that he can whip any rooster in the neighborhood, provided always that the rooster be not allowed to crow. Jim doubtless thinks he is crow enough himself, and resents any infringement upon his patent by fleeing from the ground, when a rooster essays that role.

---

THE POPULAR SCIENCE MONTHLY, published by D. Appleton & Co., New York, is a most admirable periodical, filling full a want long felt by the more intelligent people. It treats scientific subjects in a popular manner, divesting science of much of its technicalities and so bringing its study within reach of the general reader. Every household laying any claim to intelligence, in order to keep up with the times, should read this incomparable magazine.

---

STREET FRIGHTS.—In these days of numerous run-aways, it is proper that some precautions should be had with reference to the frightening of horses. A recent decision by the courts, gives the highways to the use of horses and vehicles, and not to pedestrians, flags, signs, barrels, barking dogs and processions. We are glad to know that when a gentleman's team takes fright from one of the numerous barking whiffets that are permitted the run of the streets, the owner of such canine is liable to any damage resulting from a fright to horses so incurred. Keep your dogs out of the street.

---

HOME.—And now the Summer has gone; the beautiful days and nights. The travelers in search of health and amusement have returned—the children are again at their lessons, and home-life within doors brings all together again in closer communion. Now the influence of each member of the family tells with greater force for good or evil, pleasure or pain. The cheerful smile, the manifested affection, the kind word, the helping hand, the well-timed advice come with double force in this in-door life, where all are brought in such close contact that the good or bad temper of any member of the family, from parent to babe, must affect all.

We could say much of the benefits of proper ventilation, well selected diet, exercise, etc., as aids and necessities to health, but, above all, would we urge upon parents the value of a cheerful and happy home. Instill in your children the elements of kindness and affection, give them what your purse can afford that is needed to amuse at home, and teach yourselves to drop the cares of the outer world at the threshhold of your home. You will be better able to take up the burden after the rest of the night, and you will be doubly blest in the happiness you confer by the reflected cheerfulness of home.

---

A CONTRIBUTION.—We hear of two brothers who attended church in this city, last Sunday, one of whom supplied the other with fifty cents to drop into the contribution basket. When the usher came to him, he cooly placed his hand in his pocket, exchanged the fifty cent coin for a twenty-five cent one, and dropped it into the basket, remarking to his brother on his way home, that he had reaped considerable "profit" from attending divine service for *once* in his life.

---

SPITTING.—Is there any habit more disgusting? Have you observed with what accuracy of aim a tobacco chewer will hit a flower vase ten feet distant, in the green grass, from your porch? Have you witnessed his ignoble failures too, when, from lack of sufficient pneumatic effort, he makes a miserable miss-shot, and lands the nasty gob upon your porch? We have known human beings to lie in bed and expectorate their filthiness upon the walls of the room. Have seen carpets loaded, yea, ruined with their nastiness.

Now, it occurs to us that animals never spit. They are too neat in their habits, and in this respect at least, exhibit superior breeding. A cow that doesn't seem to know much, or to speculate about matters and things, will mope about in a clover field, all day, with her mouth close to the ground, too, so that she could spit just as well as not, but she doesn't. An ass, all head and ears, with a mouth large enough to convert into a splendid spitting machine, never spits. A hog, with a long, very long snout,—suggestive of nasal catarrh—now, he could spit if he chose, and never be caught at it, for he rams his nose in the ground more than half his time, but, confound him, who ever knew of even *his* spitting? We know of no animal nasty enough to spit. They are too cleanly in their habits, and as a rule never do untidy, unseemly things. Stop your spitting!

THAT NEW YORK P. M.—They have a Postmaster in New York city that is exasperating. You can get anything under heaven *out* of his office—no matter how much the rules are violated, but nothing that can, by any possibility be construed into the slightest infringement of the Post Office laws, can you get *into* his office.

We have known Editors to copy the form of a renewal of subscription to a journal, that came through the New York office, and have letter postage charged upon their journal for sending precisely the same thing *to* New York.—Consistency is said to be a jewel. We should be glad to have that New York postmaster practise it.

---

HORSE RACING.—There is a deal of nonsense about racing. Every time we attend one, we declare it to be the last that shall entertain and disgust us. Yet we go, hoping that each one will be better conducted than its predecessor, and are, as usual, disappointed.

That everlasting scoring, in which every jockey strives to get the best start for his horse, and if he fails, runs him under the wire to be called back for another and another trial, until the audience become disgusted and tired of the performance, is a very great mar to one's pleasure. Perhaps this cannot be prevented, but it seems to be otherwise regarded by most of the gentlemen who are the patrons of the Elmira Driving Park. Then, the pool-selling is another vice that should not be permitted upon so respectable a track as the one referred to is supposed to be; besides, gentlemen, it is in direct violation of the laws of the State!

Pay more attention to the comfort and pleasure of your guests and less to the jockey's, would you have your races patronized by ladies and gentlemen.

---

A FLEA.—Last night—it must have been very late, for we were sound asleep, dreaming that "copy" was all in, and that we could have a rest among the flowers in the conservatory, when a slap came down upon our defenseless person, that made the welkin ring,—for, mind you, it was a warm night, and but little clothing adorned our prostrate and sleeping form. That slap reminded us of our younger days—of our mother, in fact, for it lighted upon the same locality as did *her* unsparing hand, when our necessities required it. We flopped over and sat upon the spot that was smarting with the blow, and inquired the cause of this miniature thunder clap. A flea! a flea! answered our spouse, as she jumped from the bed, lighted the gas, and began a careful search for the object of her terror. We soon joined in the pursuit, for, confound that flea, we owed him a grudge which we were anxious to pay. Soon we saw the little rascal perched upon the pillow,—we carefully approached him and brought our hand down upon his resting place, only to find him clear in the middle of the bed. That little cuss jumped just two hundred times his own length, at one trial! We went to speculating upon that jump, and informed our partner that if she could clear a proportionate distance, to her length, in one bound, she should be rewarded with a new gown. When she found, upon a moment's figuring, that she must jump just eleven hundred feet, she abandoned the project but informed us that, had she spanked with proportionate strength to that of the flea's, we would be now a stranger and a wanderer in a foreign land—perhaps in China. This ended our calculations for the night.

---

MY NERVOUS PATIENT.—He's very "nervous" is that patient of ours,—poor fellow, we pity him, and are at our wit's end to know how to carry him through—to allay all his fears, answer all his questions and keep him moderately contented with his lot, until at last, the day will come, when he can walk upon that poor broken leg of his.

He has his ups and downs. One day he is happy, and the next, in the very depths of despair. That which *we* consider a slightly unfavorable symptom in his case, rejoices him;—when we are jubilant over a new and expected symptom, arguing a speedy return to health and vigor again, he looks blue and cries himself into a state of nervousness that gives him a relapse of a week's duration. He eyes our every motion, when dressing that limb, and resents any new procedure with a frown and gesture akin to frenzy. So "nervous" is he, so sensitive to pain, that the prick of a pin becomes the equivalent of a gunshot wound to him. Applications that, to most person's wounds would be unfelt, to him is torture—genuine torture, too, no "make believe" sort. Hence, it is, that by reason of mild measures, lack of sleep, fretfulness, and want of exercise, our poor nervous patient is but slowly recovering,—fretting, worrying and crying his strength away that we so much needed for healing purposes. But, poor boy, he can't help it, and our heart aches for him, knowing that he has not the strength or *will* to throw off his "nervousness."

When he gets well and strong again, how we will chaff him over his fretfulness, and how he will laugh himself at his former condition and wonder how he *could* have been so very "nervous."

